# Acupuncture Analgesia in Patients With Traumatic Rib Fractures: A Randomized-Controlled Trial

**DOI:** 10.3389/fmed.2022.896692

**Published:** 2022-05-27

**Authors:** Chun-Ting Liu, Ting-Min Hsieh, Bei-Yu Wu, Yu-Chuen Huang, Chun-Han Shih, Wen-Long Hu, Ming-Yen Tsai, Yung-Hsiang Chen

**Affiliations:** ^1^Department of Chinese Medicine, Kaohsiung Chang Gung Memorial Hospital and Chang Gung University College of Medicine, Kaohsiung, Taiwan; ^2^Graduate Institute of Integrated Medicine, School of Chinese Medicine, College of Chinese Medicine, Research Center for Chinese Medicine & Acupuncture, China Medical University, Taichung, Taiwan; ^3^Department of Chinese Medicine, Dali Branch, Jen-Ai Hospital, Taichung, Taiwan; ^4^Division of Trauma, Department of Surgery, Kaohsiung Chang Gung Memorial Hospital and Chang Gung University College of Medicine, Kaohsiung, Taiwan; ^5^Fooyin University College of Nursing, Kaohsiung, Taiwan; ^6^Department of Medical Research, China Medical University Hospital, Taichung, Taiwan; ^7^Department of Psychology, College of Medical and Health Science, Asia University, Taichung, Taiwan

**Keywords:** traumatic rib fracture, acupuncture, laser acupuncture, low level laser therapy, acupuncture analgesia

## Abstract

Pain management for traumatic rib fracture is important to prevent complications and reduce associated comorbidities. This trial investigated the analgesic efficacy of acupuncture on traumatic rib fracture. Patients with traumatic rib fracture were randomly assigned to traditional acupuncture (TA), laser acupuncture (LA) or sham laser acupuncture (SLA) groups in a 1:1:1 ratio. The intervention was performed on days 1 to 3 after treatment allocation. The acupoints included bilateral LI4 (Hegu), SJ6 (Zhigou), ST36 (Zusanli) and GB34 (Yanglingquan). The primary outcome was Numeric Rating Scale (NRS) scores for pain after the intervention. Secondary outcomes included sustained maximal inspiration (SMI) lung volume, stress responses, the use of analgesics, and associated complications. Data were analyzed via one-way analysis of variance (ANOVA) with Scheffé’s *post hoc* testing or chi-squared testing. Of the 120 study participants, 109 completed all interventions and measurements. The primary outcomes, which indicated average pain intensity levels and pain while deep breathing, were both significantly lower in the TA and LA groups than in the SLA group after 2 treatments. No between-group differences were observed in SMI lung volume, stress response, analgesics use or associated complications. These findings suggest that TA and LA are safe and effective analgesic modalities for pain management for traumatic rib fracture.

**Clinical Trial Registration:**

[ClinicalTrials.gov], identifier [NCT03822273].

## Introduction

Blunt thoracic trauma can damage vital structures in the chest wall and thereby cause rib fracture, hemothorax, pneumothorax, pulmonary contusion, lung laceration, and blunt cardiac injury (1). Among all of them, rib fracture is the most common injury. In United States hospitals, an estimated 150,000–300,000 patients per year seek treatment for this injury, and mortality rates of up to 10% are reported (2). The chest wall pain induced by thoracic trauma can impair patients’ ability to clear airway secretions by coughing and deep breathing, increasing the risk of pneumonia, and respiratory failure (2). The major goal in the treatment of patients with rib fracture is to reduce pain. Appropriate analgesia and early aggressive care appear to attenuate the development of pulmonary complications. Commonly used oral analgesics such as acetaminophen and non-steroidal anti-inflammatory drugs (NSAIDs) seem to provide limited relief from severe pain (3). For years, narcotic analgesics have remained the main therapy for pain management in patients with traumatic rib fracture (4). However, narcotic regimens are often associated with multiple adverse effects, such as respiratory depression, confusion, dizziness, nausea, vomiting, and constipation. Opioid-induced respiratory depression in patients with traumatic rib fracture is lethal, especially in the presence of pneumothorax and hemothorax. Regional analgesic techniques such as epidural analgesia, thoracic paravertebral block, and intercostal nerve block also have been used for pain management, but there are many limiting factors, such as infection at the site of injection, allergy to local anesthetic drug, coagulopathy, and bleeding disorders (5). In addition, epidural analgesia is contraindicated in patients with hypotension and may increase pulmonary complications (5). Thoracic paravertebral block and intercostal nerve block entail the risks of pneumothorax and vascular puncture (5). Surgery with internal fixation for rib fracture is another effective option for relieving pain, particularly in patients with severe pain and flail chest (6). However, surgical stabilization of rib fracture is costly and may not be cost-effective for patients without flail chest (7). Currently, the optimal analgesic modality remains unknown in adults with blunt thoracic trauma including traumatic rib fracture (3). More cost-effective and harmless treatments for pain management in patients with traumatic rib fracture should be discovered.

Acupuncture is an ancient Chinese medical technique that is commonly used for pain relief. Although the mechanisms of acupuncture are not completely understood, clinical trials of acupuncture treatment increasingly suggest that acupuncture is effective for musculoskeletal pain and even cancer pain (8). The American College of Physicians suggests that acupuncture can be selected as initial non-pharmacologic treatment for the treatment of acute or chronic low back pain (9). Currently, few studies have examined acupuncture treatment for traumatic rib fracture. Of these studies, only one was a randomized controlled study; in that study, 58 patients with rib fracture experienced significant pain relief with acupuncture as compared with controls (10).

The purpose of this study was to investigate the analgesic efficacy of acupuncture for traumatic rib fracture. The hypothesis of the study was that acupuncture could alleviate the pain associated with traumatic rib fracture, improve respiration, and prevent pneumonia.

## Materials and Methods

### Ethics Approval

The protocol was registered with ClinicalTrials.gov (Identifier: NCT03822273). The study was approved by the Human Ethics Committee of Chang Gung Medical Foundation Institutional Review Board, IRB No. 201701455A3. This study was conducted in accordance with the principles of the Declaration of Helsinki. Both verbal and written forms of detailed information about the trial were provided before participation by trauma surgery physicians at Kaohsiung Chang Gung Memorial Hospital (KCGMH). All participants voluntarily signed and provided informed consent that had been approved by the ethics committee prior to enrollment. The participants received no financial benefit from the study and were fully aware of their rights to withdraw at any time. Personal information about potential and enrolled participants was collected, shared, and maintained in an independent and secure storage space to protect the participants’ confidentiality before, during, and after the trial.

### Study Design

**FIGURE 1:**
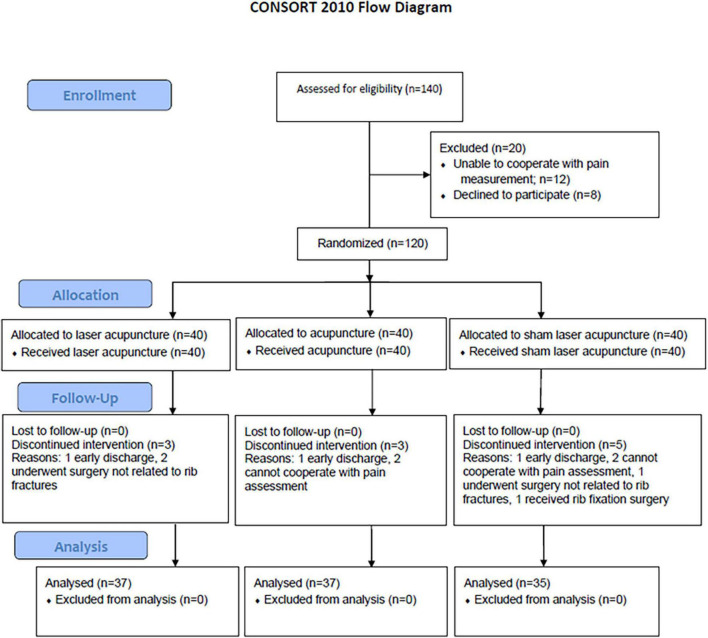
The flowchart of the trial.

This single-center, prospective, randomized controlled clinical trial (RCT) was conducted from January 2018 to February 2020 at KCGMH, a 2,600-bed tertiary medical center with an average of 9,000 emergency department visits per month. Patients with rib fracture hospitalized in the ward of Trauma Surgery were recruited. Patients with rib fracture who wanted to participate in the study were screened for eligibility through a chart review and interview on the first visit. The participants who met the inclusion criteria were randomly assigned to true acupuncture (TA), laser acupuncture (LA), or sham laser acupuncture (SLA) groups in a 1:1:1 ratio. The study design, developed in accordance with the Consolidated Standards of Reporting Trials (CONSORT) 2010, is depicted in Figure 1.

### Eligibility Criteria

Participants’ eligibility to participate in the study was assessed by trauma surgeons. Written informed consents were obtained for all participating patients before randomization. All the patients with traumatic rib fracture were managed from the time of admission by the trauma surgery team, who had no information about the allocation results.

The study inclusion criteria were as follows: (1) aged 20 years or older; (2) presence of one or more, unilateral or bilateral rib fractures diagnosed with chest X-ray; (3) maximal rib pain score of more than 5 on the numerical rating scale (NRS: 0–10) while doing any one of the following three actions: deep breathing, coughing, or turning over; and (4) ability to describe the sites of pain and evaluate the intensity accurately.

The participants with the following conditions were excluded: (1) inability to be examined for the movements of deep breathing, coughing, or turning over in bed; (2) local skin infection or open wound on the acupoints, or limb amputees; (3) cognitive impairment that precluded the accurate clinical assessment of the NRS score; (4) severe multiple trauma or any poorly controlled diseases such as atelectasis, pneumonia, or other infectious diseases, immune system dysfunction, bleeding tendency, psychiatric disorders, and skin problems; (5) communication difficulties; (6) surgical stabilization of new rib fracture before enrollment; (7) unwillingness to provide informed consent; or (8) determination of unsuitability for this study by medical staff. In addition, participants would be dropped from the trial under the following conditions: (1) unstable vital signs and/or need for first aid during study period; (2) surgical stabilization of rib fracture during study period; and (3) voluntary decision by participants to withdraw from the trial at any time.

### Sample Size Calculation, Blinding, and Randomization

Based on a previous study (10) on the effectiveness of acupuncture in patients with rib fracture, the mean ± standard deviation of the NRS scores on day 3 after treatment were 1.3 ± 1.4 in the acupuncture group and 2.6 ± 0.5 in the control group. Anticipating a power of 80% (1 – β = 0.8), statistical significance of 95% (α = 0.05), and a dropout rate of 20%, a total of 50 participants were required in each group, according to G*power analysis.

All the participants were told that they would receive one kind of acupuncture treatment in addition to the conventional treatment. The participants were not notified about which group they were allocated to or which kind of acupuncture treatment would be applied as a control before randomization. Enrolled patients were randomly assigned in a ratio of 1:1:1to the TA, LA, and SLA groups for three parallel treatments. Randomization was conducted using block randomization with a block size of 3 for the three groups. Randomization was executed by an independent researcher, who was not involved in the inclusion or exclusion process, treatment, or assessment procedures. Participants randomized to the LA or SLA groups were blinded to laser acupuncture. The outcome assessor was blinded to the group assignments and was well trained in the use of the pain scale and physical tests. To avoid unblinding of the assessment of outcomes, the participants were instructed not to discuss any aspect of their treatment with the assessor.

### Intervention

Regardless of treatment group, the acupuncture regimen included stimulation of the same acupoints by a certified traditional Chinese medicine (TCM) physician, who was also the only person in this trial aware of the group assignment. All participants received conventional pain management offered by trauma surgeons. Drugs used for underlying chronic diseases were not restricted.

### Traditional Acupuncture Group

**FIGURE 2:**
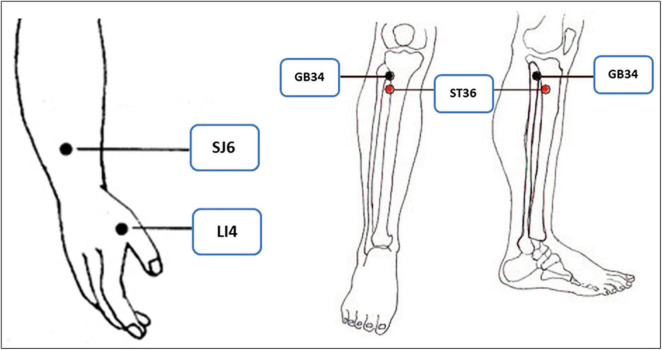
The acupoints selected in the trial.

Participants in the TA group were treated with filiform needles once daily for three consecutive days after the day of enrollment. Eight needles were inserted at the following acupoints: the bilateral LI4 (Hegu), SJ6 (Zhigou), ST36 (Zusanli), and GB34 (Yanglingquan) (Figure 2), which were located according to the WHO Standardized Acupuncture Point Location guidelines (11). Disposable sterilized stainless-steel needles were inserted to a depth of 15–35 mm. All needles were rotated manually at least once at each session to elicit the needle sensation (*de-qi*). The needle retention time was 15 min.

### Laser Acupuncture Group

Participants allocated to the LA group received laser acupuncture treatment at the same acupoints used in the TA group. The laser acupuncture therapy was performed with a gallium aluminum arsenide LaserPen [maximal power, 150 mW; wavelength, 810 nm; area of probe, 0.03 cm^2^; power density, 5 W/cm^2^; pulsed wave; and Bahr frequencies (B1: 599.5 Hz, B2: 1199 Hz, B3: 2398 Hz, B4: 4776 Hz, B5: 9552 Hz, B6: 19,104 Hz, and B7: 38,208 Hz); RJ-Laser, Reimers & Janssen GmbH, Waldkirch, Germany]. The laser was applied to each point for 5 s and delivered 0.375 J of energy at each of the acupoints.

### Sham Laser Acupuncture Group

Participants in the SLA group underwent sham laser acupuncture treatment without any laser output. The acupuncture points, application duration, and total number of treatments were the same as those in the LA group.

### Study Outcome

The primary outcome measure was the NRS score for pain on days 1–3 after treatment. The scores of NRS for pain were assessed on average and while deep breathing, coughing, and turning over. Since pain is a subjective experience, the exact quality and quantity of pain are very difficult for people to express, and no diagnostic tests are available for assessing pain. A pain scale such as the NRS is thus a relatively objective tool to measure and describe the pain of an individual. The NRS is a simple tool for verbally describing pain intensity on a scale of 0–10 points, where 0 = no pain and 10 = worst possible pain.

The secondary outcome included sustained maximal inspiration (SMI) lung volume measured by incentive spirometer, stress response, the use of narcotic and non-narcotic medications, and the use of laxatives, antacids, and proton pump inhibitors during the study period. The number of participants who used these drugs was calculated during the intervention on days 1–3 and after the intervention on day 4. The stress responses associated with pain were measured from salivary cortisol level, heart rate variability (HRV), heart rate, mean arterial pressure and sleep quality (0–10; 0 = no problem, 10 = sleep totally interrupted by pain) on days 1–3 after treatment. Finally, the complications of pneumonia and gastrointestinal (GI) bleeding within one month were recorded after enrollment.

Salivary samples were obtained from the participants before the intervention and on days 1–3 after the intervention. After collection, the samples were kept cold in order to prevent bacterial growth in the specimens. The samples were immediately refrigerated at 4°C and then, within 4 h of collection, stored at −20°C until analysis. Prior to analysis, the samples were thawed completely, vortexed, and centrifuged at 1,500 × *g* for 15 min to remove mucins and other particulate matter which could interfere with the assay and affect the results. Salivary cortisol activity was determined using a Salimetrics^®^ Cortisol Enzyme Immunoassay Kit (Item No. 1-3002, Salimetrics LLC, State College, PA, United States), which is a competitive immunoassay used to quantitatively measure salivary cortisol levels. The cortisol in standards and samples compete with cortisol conjugated to horseradish peroxidase for the antibody binding sites on a microtiter plate. According to the manufacturer’s protocol, after incubation, unbound components were washed away, and bound cortisol enzyme conjugate was measured by the reaction of horseradish peroxidase enzyme to the substrate tetramethylbenzidine. This reaction produces a blue color, and a yellow color is formed after the reaction is stopped with an acidic solution. The optical density was read on a standard plate reader at 450 nm.

### Statistical Analysis

Both intention-to-treat (ITT) analysis using all available data and per-protocol (PP) analysis using only data from participants who completed all treatment sessions were performed. The clinical characteristics data were analyzed via one-way analysis of variance (ANOVA) with Scheffé’s *post hoc* testing or chi-squared testing, as appropriate. Quantitative variables, presented as mean ± SD, were analyzed by ANOVA with Scheffé’s *post hoc* testing. Qualitative variables, expressed as a number (percentage), were analyzed using the Chi-Squared test. Lengths of hospital stay in participants with or without comorbid clavicle fracture in each group were analyzed by independent *t*-tests. All analyses were performed in SPSS 22.0 for Windows (Statistics 22.0, SPSS, IBM, New York, NY, United States). Differences were considered statistically significant at *P* < 0.05.

## Results

### Patient Characteristics

As shown in the flowchart of the trial in Figure 1, 140 patients with rib fracture were assessed for eligibility and 120 patients met the criteria. Of the 120 study participants, 109 completed all interventions and measurements.

One patient in each of the TA, LA, and SLA groups was discharged from the hospital early and could not receive complete treatment and assessment. Two patients in each of the TA and SLA groups could not cooperate with pain assessment. Two patients in the LA group and one in the SLA group stopped treatment due to surgery unrelated to rib fracture. Another patient in the SLA group dropped out due to surgical stabilization of the rib fracture. The PP and ITT analyses of the clinical characteristics of the participants showed consistent results. The results of the PP and ITT analyses are listed in Table 1 and Supplementary Table 1, respectively, and statistically significant differences were found. The factors associated with pain intensity, including number of ribs fractured and injury severity scores, were not significantly different between groups.

**TABLE 1:** Clinical characteristics of participants in the study.

Characteristics	Acupuncture (*N* = 37)	Laser acupuncture (*N* = 37)	Sham Laser acupuncture (*N* = 35)	*P* value
Age, years, mean ± SD^a^	54.49 ± 15.81	54.46 ± 15.87	54.06 ± 13.59	0.991
Male gender, n (%)^b^	22 (59.5%)	27 (73%)	20 (57.1%)	0.317
BMI (kg/m^2^)^a^	26.15 ± 5.28	26.38 ± 5.33	24.27 ± 3.46	0.134
Current smoker, n (%)^b^	12 (32.4%)	11 (29.7%)	10 (28.6%)	0.800
Mechanism of injury, n (%)^b^				0.444
Traffic accident	31(83.8%)	27 (73.0%)	25 (71.4%)	
Fall	1 (2.7%)	5 (13.5%)	5 (14.3%)	
Crush	5 (13.5%)	5 (13.5%)	5 (14.3%)	
Number of ribs fractured^a^	4.05 ± 2.26	4.19 ± 2.07	3.89 ± 1.84	0.821
Injury Severity Score^a^	10.95 ± 5.59	9.97 ± 4.32	11.94 ± 6.80	0.337
Complications, n (%)^b^				0.950
Pneumothorax	6 (16.2%)	6 (16.2%)	4 (11.4%)	
Hemothorax	3 (8.1%)	4 (10.8%)	3 (8.6%)	
Hemopneumothorax	1 (2.7%)	3 (8.1%)	2 (5.7%)	
Chest tube/pig tail insertion, n (%)^b^	4 (10.8%)	3 (8.1%)	5 (14.3%)	0.694
Trauma to admission (days)^a^	1.86 ± 1.72	1.54 ± 0.77	1.66 ± 0.97	0.517
Admission to intervention (days)^a^	4.43 ± 2.32	4.05 ± 1.89	4.17 ± 1.86	0.716
Admission to discharge (days)^a^	9.3 ± 3.3	10.6 ± 5.6	10.8 ± 4.8	0.352

*^a^One-way analysis of variance with Scheffé’s post hoc testing. ^b^Chi-squared testing. SD, standard deviation; BMI, body mass index.*

### Primary Outcome

**FIGURE 3:**
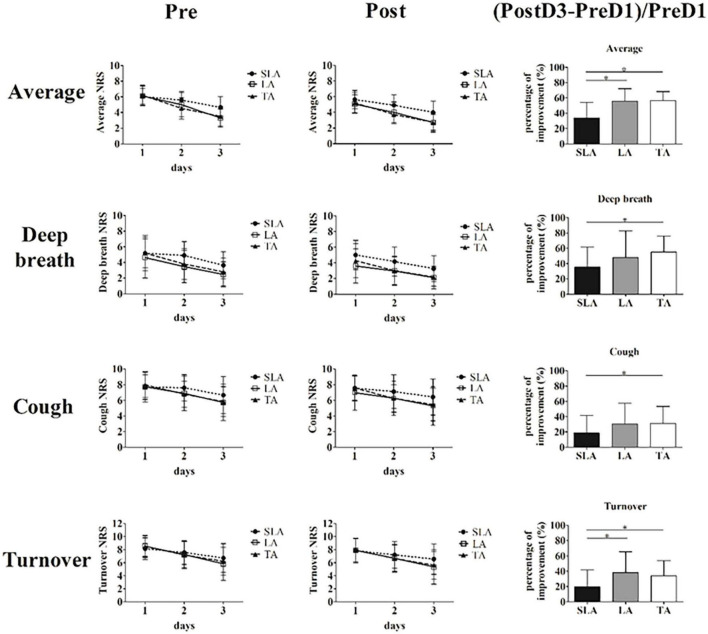
Primary efficacy variables, and distributions of average pain level and pain while deep breathing, coughing and turning over before and after treatment. (Data of (Post3-Pre1)/Pre1 were analyzed via one-way analysis of variance with Scheffé’s *post hoc* testing. *Significance when compared with sham laser acupuncture group, *p < 0.05. Pre, before treatment; Post, after treatment; SLA, sham laser acupuncture; LA, laser acupuncture; TA, acupuncture).

The PP and ITT analyses of the primary outcome also showed consistent results. The NRS scores for pain intensity on days 1 to 3 after treatment using PP and ITT analyses are listed in Table 2 and Supplementary Table 2. The initial average pain intensity levels and pain while coughing, turning over and deep breathing were not significantly different between groups before treatment. Average pain intensity levels and pain while deep breathing were both significantly lower in the TA and LA groups than in the SLA group after two treatments. There were no significant differences in the NRS scores while coughing and turning over after treatment between groups. The primary efficacy variables were the percentage changes in NRS scores between baseline and on day 3 after treatment. The distribution of NRS scores before and after treatment and the statistics for the percentage changes in NRS scores between groups are presented in Figure 3. According to the NRS scores on day 3, the percentage improvement was significantly higher in the TA group than in the SLA group in terms of average pain intensity levels, pain while coughing, turning over and deep breathing. The percentage improvements in average pain intensity and pain while turning over were significantly higher in the LA group than in the SLA group.

**TABLE 2:** NRS scores for pain intensity before and after treatment on days 1–3.

NRS	Intervention day	Acupuncture	Laser acupuncture	Sham laser acupuncture	*P* value
Average	pre D1	6.19 ± 1.29	6.11 ± 1.27	6.06 ± 1.00	0.894
	post D1	5.22 ± 1.29	5.05 ± 1.20	5.63 ± 1.17	0.128
	post D2	3.73 ± 1.19*	4.00 ± 1.31*	4.91 ± 1.34	<0.001
	post D3	2.73 ± 1.02*	2.73 ± 1.24*	4.00 ± 1.44	<0.001
Deep	pre D1	5.19 ± 2.26	4.62 ± 2.62	5.17 ± 1.86	0.480
breath	post D1	4.27 ± 2.18	3.59 ± 2.18*	5.00 ± 1.88	0.020
	post D2	3.00 ± 1.78*	2.97 ± 1.87*	4.11 ± 1.86	0.013
	post D3	2.19 ± 1.18*	2.11 ± 1.45*	3.26 ± 1.67	0.001
Cough	pre D1	7.97 ± 1.61	7.73 ± 1.95	7.69 ± 1.57	0.745
	post D1	7.57 ± 1.54	6.97 ± 2.24	7.54 ± 1.62	0.293
	post D2	6.24 ± 1.75	6.27 ± 2.19	7.11 ± 2.14	0.127
	post D3	5.46 ± 2.09	5.27 ± 2.47	6.43 ± 2.32	0.078
Turnover	pre D1	8.49 ± 1.69	8.54 ± 1.56	8.14 ± 1.67	0.541
	post D1	7.92 ± 1.82	7.89 ± 1.79	7.86 ± 1.90	0.990
	post D2	6.65 ± 2.12	6.68 ± 2.00	7.20 ± 2.06	0.449
	post D3	5.62 ± 2.13	5.35 ± 2.65	6.54 ± 2.33	0.090

*NRS, Numerical Rating Scale; pre D1, NRS score on day 1 before treatment; post D1, D2, D3, NRS score on day 1–3 after treatment.*

*Significance when compared with sham laser acupuncture group, *P < 0.05.

### Secondary Outcome

The data on the secondary outcome were analyzed using PP analysis. No between-group differences were observed in SMI lung volumes, salivary cortisol levels, HRV, heart rate, mean arterial pressure or sleep quality on days 1–3 after treatment (Table 3). The use of narcotic and non-narcotic medications for pain between groups was not significantly different during and after intervention (Table 4). The use of laxatives, antacids and PPIs for GI disorders are presented in Table 5, showing that the use of laxatives between groups was not significantly different during and after intervention. In contrast, the use of antacids and PPIs was significantly lower in the TA group and higher in the SLA group than in the LA group after intervention. The participants in the SLA group tended to use more laxatives, antacids and PPIs during the intervention, but the difference was not statistically significant. One patient in each of the TA and LA groups had pneumonia during hospitalization. One patient in each of the TA and LA groups had GI bleeding within the one-month follow-up period. No between-group differences were observed for the complications of pneumonia and GI bleeding (Table 5). The subgroup analysis of the length of hospital stay for participants with or without comorbid clavicle fracture is presented in Table 6. Overall, patients with combined rib and clavicle fracture had significantly longer hospital stays than those with rib fracture alone (12.3 ± 5.2 days vs. 9.6 ± 4.4 days; *P* = 0.011). This phenomenon was similar in the SLA group (15.8 ± 3.6 days vs. 9.8 ± 4.4 days; *P* = 0.003), but inconsistent with the TA group (9.9 ± 5.0 days vs. 9.1 ± 2.4 days; *P* = 0.645) and LA group (12.6 ± 5.1 days vs. 9.9 ± 5.7 days; *P* = 0.225). In comparison between groups, however, the patients with combined rib and clavicle fracture in the SLA group had longer hospital stays, but there was no statistically significant difference from the TA and LA groups.

**TABLE 3:** SMI volume, pain-induced stress responses before and after treatment.

	Intervention day	Acupuncture	Laser acupuncture	Sham laser acupuncture	*P* value
SMI	pre D1	627.0 ± 277.3	668.9 ± 219.3	658.0 ± 220.6	0.741
	post D1	773.0 ± 213.6	767.6 ± 208.6	758.6 ± 214.0	0.959
	post D2	914.9 ± 241.8	909.5 ± 211.8	848.6 ± 217.8	0.386
	post D3	944.6 ± 237.6	931.6 ± 229.1	917.1 ± 212.5	0.881
Cortisol	pre D1	0.26 ± 0.14	0.41 ± 0.30	0.38 ± 0.35	0.067
	post D1	0.21 ± 0.12	0.21 ± 0.21	0.20 ± 0.12	0.951
	post D2	0.16 ± 0.09	0.22 ± 0.13	0.17 ± 0.08	0.037
	post D3	0.20 ± 0.18	0.23 ± 0.17	0.23 ± 0.17	0.761
LF	pre D1	998.5 ± 1506.4	416.9 ± 557.7	1162.8 ± 2185.6	0.135
	post D1	1248.5 ± 1718.5	844.0 ± 1512.5	767.3 ± 1314.4	0.387
	post D2	2013.0 ± 4015.1	988.0 ± 1805.6	991.2 ± 1414.2	0.191
	post D3	1170.7 ± 1701.2	1057.3 ± 2474.8	1123.8 ± 1958.5	0.947
HF	pre D1	626.3 ± 691.0	433.78 ± 573.0	560.2 ± 648.6	0.438
	post D1	960.4 ± 1383.0	533.2 ± 674.4	593.6 ± 804.2	0.167
	post D2	766.5 ± 795.3	673.6 ± 879.2	729.5 ± 809.9	0.894
	post D3	773.1 ± 896.2	602.7 ± 779.6	600.1 ± 712.2	0.595
LF/HF	pre D1	1.42 ± 1.20	1.20 ± 0.87	1.57 ± 1.76	0.527
	post D1	1.32 ± 0.90	1.36 ± 1.23	1.32 ± 1.62	0.990
	post D2	1.71 ± 1.83	1.42 ± 1.30	1.55 ± 1.14	0.694
	post D3	1.39 ± 0.96	1.55 ± 1.56	1.84 ± 1.17	0.361
HR	pre D1	77.6 ± 13.3	76.8 ± 11.9	76.5 ± 13.0	0.935
	post D1	75.4 ± 13.0	76.3 ± 12.0	75.1 ± 11.1	0.919
	post D2	79.1 ± 12.0	76.7 ± 10.7	77.7 ± 12.5	0.696
	post D3	78.2 ± 11.7	76.8 ± 10.0	76.9 ± 11.2	0.842
MAP	pre D1	91.4 ± 14.7	100.8 ± 15.0	97.6 ± 18.1	0.124
	post D1	92.2 ± 14.0	100 ± 17.7	93.3 ± 17.6	0.227
	post D2	97.4 ± 15.4	95.8 ± 28.4	95.9 ± 17.5	0.959
	post D3	95.3 ± 12.8	95.4 ± 24.0	95.9 ± 12.3	0.991
Sleep	pre D1	6.1 ± 1.8*	5.6 ± 1.8	4.9 ± 2.3	0.048
	post D1	6.0 ± 2.1	5.4 ± 2.6	5.7 ± 2.7	0.586
	post D2	5.4 ± 2.9	5.5 ± 2.8	5.8 ± 2.3	0.824
	post D3	5.1 ± 2.8	6.5 ± 2.1	6.7 ± 2.0	0.052

*pre D1, NRS score on day 1 before treatment; post D1, D2, D3, NRS score on day 1 to 3 after treatment; NRS, Numerical Rating Scale; SMI, sustained maximal inspiration; LF, low frequency; HF, high frequency; LF/HF, LF/HF ratio; HR, heart rate; MAP, mean arterial pressure.*

*Significance when compared with sham laser acupuncture group, *P < 0.05.

**TABLE 4:** The use of narcotic and non-narcotic medications during intervention (days 1–3) and after intervention (day 4).

	Intervention day	Acupuncture (37), *n* (%)	Laser acupuncture (37), *n* (%)	Sham laser acupuncture (35), *n* (%)	*P* value
Opioids	During	30 (81.1%)	29 (78.4%)	26 (74.3%)	0.783
	After	16 (43.2%)	14 (37.8%)	17 (48.6%)	0.655
NSAIDs	During	22 (59.5%)	24 (64.9%)	21 (60.0%)	0.871
	After	12 (32.4%)	10 (27.0%)	8 (22.9%)	0.659
Acetaminophen	During	9 (24.3%)	11 (29.7%)	6 (17.1%)	0.455
	After	5 (13.5%)	4 (10.8%)	4 (11.4%)	1.0

*NSAIDs, non-steroidal anti-inflammatory drugs.*

**TABLE 5:** The use of laxatives and antacid/PPI during intervention (days 1–3) and after intervention (day 4), and complications during hospitalization and follow-up for one month.

	Intervention day	Acupuncture (37), n (%)	Laser acupuncture (37), n (%)	Sham laser acupuncture (35), n (%)	*P* value
Laxatives	During	13 (35.1%)	13 (35.1%)	20 (57.1%)	0.095
	After	3 (8.1%)	6 (16.2%)	9 (25.7%)	0.132
Antacid/PPI	During	19 (51.4%)	20 (54.1%)	27 (77.1%)	0.050
	After	5* (13.5%)	16* (43.2%)	10 (28.6%)	0.018
Pneumonia	During hospitalization	1 (2.7%)	1 (2.7%)	0 (0%)	1.000
	f/u 1 month	–	–	–	–
GI bleeding	During hospitalization	–	–	–	–
	f/u 1 month	1 (2.7%)	1 (2.7%)	0 (0%)	1.000

*PPI, proton pump inhibitor; GI, gastrointestinal.*

**TABLE 6:** Length of hospital stay in participants with or without combined clavicle fracture.

	Acupuncture, day (*n*)	Laser acupuncture, day (*n*)	Sham laser acupuncture, day (*n*)	*P*^#^ value	All patients; day (*n*)
Rib fractures	9.1 ± 2.4 (27)	9.9 ± 5.7 (28)	9.8 ± 4.4 (29)	0.769	9.6 ± 4.4 (84)
Combined clavicle fracture	9.9 ± 5.0 (10)	12.6 ± 5.1 (9)	15.8 ± 3.6 (6)	0.076	12.3 ± 5.2 (25)
P* value	0.645	0.225	0.003		0.011

*^#^One-way analysis of variance with Scheffé’s post hoc testing.*

*Independent t-tests.

## Discussion

Currently, acupuncture is increasingly used for a variety of diseases worldwide. Although considerably large acupuncture research had demonstrated that acupuncture is safe for many conditions in the past two decades (12, 13), there is still no standard protocol for pain management. The main reason is that there are various types and protocols of acupuncture treatment for one condition or disease. There is currently no standard approach or prescription for acupuncture treatment. Most of the acupoints used in acupuncture treatment are based on the experience of physicians, and these treatments often lack clinical trials to verify their efficacy. Therefore, well-designed randomized controlled trials with a widely accepted protocol of acupuncture treatment are still needed. To the best of our knowledge, few publications have described acupuncture for traumatic rib fracture. Only one randomized controlled trial (10) has demonstrated a positive effect on acute pain using a novel acupuncture modality. In that study, the needle insertion sites were on the abdomen or back and not on classical acupoints. Although this novel acupuncture modality is safe, it lacks the theoretical basis of TCM, and the selected needle insertion sites are rarely used in real acupuncture practice.

The present study is the first RCT to investigate acupuncture analgesia using classical acupoints in patients with traumatic rib fracture. The main results of our study showed that the average pain intensity levels of pain while coughing, turning over and deep breathing were all significantly lower in the TA group than in the SLA group, and the average pain intensity and pain while turning over were both significantly lower in the LA group than in the SLA group. The acupuncture modality in our study is commonly implemented and can be widely accepted. The selected acupoints, namely, LI4 (Hegu), SJ6 (Zhigou), ST36 (Zusanli), and GB34 (Yanglingquan), are based on the theory of TCM. All acupoints are located on the extremities, which are completely safe for needle insertion. According to meridian theory, the acupoints LI4 and ST36, belonging to the yang-ming meridian, and SJ6 and GB34, belonging to the shao-yang meridian, can potentially improve *qi* and blood flow and subsequently alleviate pain on the front and lateral areas of the chest, respectively. In acupuncture clinical practice, LI4 is a very common and useful point for any condition related to the face and head, while ST36 is typically used to treat GI disturbance. Combining these two acupoints can produce a good analgesic effect anywhere in the body (14, 15). SJ6 is typically used for constipation and pain in the lateral costal region, ribs and axilla. GB34 is typically used for motor disorders and pain (16, 17), particularly those associated with trauma and in the lateral side of the body.

Regarding clinical trials of acupuncture treatment, an appropriate design for control group and double-blinding procedure remains a methodological challenge. The commonly used controls in acupuncture trials include non-intervention control, non-insertion sham control, and needle insertion at sham or real acupoints (18). Although non-insertion sham control such as non-penetrating devices resembles the real acupuncture needling procedure, patients are very likely to distinguish between real and fake acupuncture because of the lack of needling sensations, such as numbness, soreness, distention, or dull pain, in the sham controls. Another sham needle insertion control using wrong points or superficial penetration may produce non-specific effects of blood circulation and neural stimulation, such as non-specific endorphin release (18, 19). Accordingly, no reliable blinding methodology for needle acupuncture has been achieved so far. Recently, SLA has been used as a control for needle acupuncture trials. It is reported that SLA can serve as a valid placebo control in LA studies due to their similar credibility and the lack of sensory input on the peripheral nervous system, and it can serve as a sham control for acupuncture trials when there is a need to evaluate the effects of needling *per se* (20). A 3-arm parallel randomized trial with TA, LA and SLA was conducted in our study. SLA treatment affords all requirements to produce the same non-specific effects as LA treatment, which can verify the efficacy of the selected acupoints in the study. SLA can serve as a sham procedure, and LA can serve as an active comparator for evaluating the needling effect of real acupuncture. We suggest that it is currently the best of all designs that can both confirm the specific effect of acupuncture and verify the specificity of selected acupoints.

All the participants we enrolled needed to be hospitalized. Twice as many participants as in the previous study were enrolled in our study. The intervention timing in this study was when the patient could not obtain adequate pain control after conventional analgesics. This means that the participants in our study had moderate injuries and uncontrollable pain. During this period, surgeons often need to face some questions, including whether to increase the dose of analgesics, increase other alternative methods of pain relief, or recommend surgical fixation. The present study aimed to investigate the immediate analgesic effect of adjuvant acupuncture in the treatment of traumatic rib fracture. Both PP and ITT analyses were performed on the clinical characteristics and primary outcome. The PP and ITT analyses of the data were consistent in this study. Additionally, none of the participants dropped out of the trial due to rib pain worsening. We considered that it was more important to ensure that the participants received complete treatments than to investigate the effect of acupuncture as an add-on therapy. Therefore, information with PP analysis should be sufficient from the trial.

According to the primary outcome in the study, the analgesic effect appeared to be significant after 2 treatments in the TA and LA groups. This finding suggests that acupuncture can provide good pain relief in about two days during hospitalization. In addition, according to the percentage improvement of the NRS scores in average and pain-inducing movements, the analgesic effect appeared to be greater with TA than with LA. A possible explanation is that TA induces *de-qi* sensation, a distinct skin insertion associated with gentle twisting or up-and-down movement, which can induce more nociceptive stimulus to modulate pain signals than can LA. LA does not produce sensation; however, it has photochemical reactions in cells and provokes nerve fiber activation, resulting in physiologic changes similar to TA (20). Our research results demonstrated that acupuncture is an effective non-pharmacologic treatment for pain management in this period. We think that if the pain control is still insufficient after a patient receives acupuncture treatment for 3 days, surgical fixation may be a good choice.

In this study, we hypothesized that acupuncture could reduce pain associated with rib fracture and subsequently improve respiration and prevent the complication of pneumonia. The results showed that acupuncture had a good analgesic effect for rib fracture, but it did not significantly increase the maximal inspiration lung volume or decrease pneumonia compared to the controls. One possible reason is that the participants enrolled in the study were those with stable vital signs without dyspnea during the study period. This factor might decrease the beneficial effects of acupuncture for pulmonary complications associated with rib fracture.

Other secondary outcomes assessing acute stress responses, including salivary cortisol levels, heart rate, HRV, mean blood pressure and sleep disorder, showed no significant differences between groups. There are several possible reasons for these results. First, the average pain levels and pain while deep breathing were reduced from moderate to mild, and the pain while coughing and that while turning over were reduced from severe to moderate. Both results show that the pain was not completely improved after full treatment and might still have caused stress responses. Another might be that the psychological impact of the injuries had been sustained for a long time (21), so these stress responses were unable to improve quickly even if the pain was under control.

According to the general perception of pain management, the use of analgesic medicines will be decreased as the pain improves. In contrast to one previous systemic review and meta-analysis (22), which showed that acupuncture could improve acute postoperative pain and reduce opioid use, our results showed that the use of narcotic and non-narcotic medications did not differ significantly between groups during and after intervention. We believe that the reason is mainly due to the short duration of our intervention and different mechanisms of the injuries. In the literature, the duration of pain after rib fracture is considered to be at least 8 weeks (23). Physicians would not stop the analgesics so quickly, even if the patient’s pain had been improved during a short-term three-day treatment in our study.

Notably, we found an interesting phenomenon, namely, that participants in the TA and LA groups had lower tendencies to use laxatives and antacids/PPI during the intervention as compared to the SLA group, but without statistical significance. Significantly fewer participants in TA group and more participants in LA group used antacid/PPI after intervention as compared with the SLA group. We think that the benefits on GI disorders mainly come from acupuncture at the ST36 point (24), and the effect of TA lasts longer than that of LA because real needling effect.

GI bleeding is another complication associated with acute stress and the use of NSAIDs. The events of GI bleeding during hospitalization and follow-up for one month showed no significant differences between groups in our study. Finally, we also analyzed the lengths of hospital stay between subgroups based on whether participants had clavicle fracture in addition to rib fracture. Of all the participants, patients having traumatic rib fracture combined with clavicle fracture had significantly longer hospital stays than those of patients with traumatic rib fracture alone. This trend was observed in the SLA group but not in the TA and LA groups. This difference suggests that acupuncture may shorten hospital stays for patients with combined clavicle and rib fracture; however, this benefit is not very significant.

There are several limitations to our study. First, the single-center scope of the study and the relatively small sample size may have led to overestimation of the effects of acupuncture. Secondly, several factors may affect the measurement of HRV and sleep quality, including patients’ condition (e.g., pain), environmental factors (e.g., light and sound disturbances), or psychological factors (e.g., anxiety and stress) (25). Finally, we were unable to choose and unify the treatment timing because the participants were recruited at different times post injury. However, the results show that early acupuncture intervention within a few days of the trauma can reduce the rib pain. Further research is needed to understand the potential long-term analgesic effect in patients with traumatic rib fracture.

## Conclusion

The findings of this study suggest that TA and LA are safe and effective analgesic modalities for pain management in traumatic rib fracture. As a non-pharmacologic adjuvant treatment, acupuncture has considerable potential for aiding in pain reduction. Acute pain management following traumatic rib fracture is a common challenge, and these results can aid physicians seeking feasible and effective methods to manage pain and improve the patient experience.

## Data Availability Statement

The original contributions presented in the study are included in the article/Supplementary Material, further inquiries can be directed to the corresponding authors.

## Ethics Statement

The studies involving human participants were reviewed and approved by Human Ethics Committee of Chang Gung Medical Foundation Institutional Review Board, IRB No. 201701455A3. The patients/participants provided their written informed consent to participate in this study.

## Author Contributions

C-TL, T-MH, and W-LH: conceptualization. B-YW: data curation. B-YW and Y-CH: formal analysis. C-TL: funding acquisition, project administration, and writing—original draft. C-TL, T-MH, and C-HS: investigation. C-TL and T-MH: methodology. M-YT and Y-HC: supervision and writing—review and editing. All authors contributed to the article and approved the submitted version.

## Conflict of Interest

The authors declare that the research was conducted in the absence of any commercial or financial relationships that could be construed as a potential conflict of interest.

## Publisher’s Note

All claims expressed in this article are solely those of the authors and do not necessarily represent those of their affiliated organizations, or those of the publisher, the editors and the reviewers. Any product that may be evaluated in this article, or claim that may be made by its manufacturer, is not guaranteed or endorsed by the publisher.
