# Supplementary material for: Acupuncture Analgesia in Patients With Traumatic Rib Fractures: A Randomized-Controlled Trial
Source: Front Med (Lausanne). 2022 May 27;9:896692. doi: 10.3389/fmed.2022.896692 (PMC9197317; doi:10.3389/fmed.2022.896692)
Supplement: Supplementary file 1 [file Table_1.DOCX]

**Supplementary Table 1. Characteristics of participants in acupuncture, laser acupuncture and sham laser acupuncture groups (using intention to treat analysis)**

| Characteristics | Acupuncture  (N=40) | Laser acupuncture (N=40) | Sham Laser acupuncture (N=40) | *p* value |
| --- | --- | --- | --- | --- |
| Age, years, mean±SD | 54.28±15.67 | 54.55±15.27 | 54.30±12.87 | 0.996 |
| Male gender, n (%) | 25 (62.5%) | 29 (72.5%) | 22 (55.0%) | 0.291 |
| BMI (kg/m^2^) | 26.11±5.14 | 26.35±5.37 | 24.86±3.72 | 0.329 |
| Current smoker, n (%) | 12 (30%) | 11 (27.5%) | 10 (25%) | 0.744 |
| Mechanism of injury, n (%) |  |  |  | 0.480 |
| Traffic accident | 34 (85.0%) | 30 (75.0 %) | 30 (75.0%) |  |
| Fall | 1 (2.5%) | 5 (12.5%) | 5 (12.5%) |  |
| Crush | 5 (12.5%) | 5 (12.5%) | 5 (12.5%) |  |
| Number of ribs fractured | 4.20±2.38 | 4.20±2.04 | 3.95±1.82 | 0.827 |
| Injury Severity Score | 10.90±5.40 | 10.08±4.22 | 11.93±6.42 | 0.314 |
| Complications, n (%) |  |  |  | 0.900 |
| Pneumothorax | 7 (17.5%) | 8 (20.0%) | 5 (12.5%) |  |
| Hemothorax | 3 (7.5%) | 4 (10.0%) | 4 (10.0%) |  |
| Hemopneumothorax | 1 (2.5%) | 3 (7.5%) | 2 (5.0%) |  |
| Chest tube/pig tail insertion, n (%) | 4 (10.0%) | 3 (7.5%) | 5 (12.5%) | 0.928 |
| Trauma to admission (days) | 1.85±1.67 | 1.50±0.75 | 1.68±0.94 | 0.424 |
| Admission to intervention (days) | 4.33±2.27 | 4.08±1.87 | 4.35±2.17 | 0.813 |
| Admission to discharge (days) | 9.23±3.46 | 10.48±5.44 | 10.88±5.34 | 0.285 |

1. All data are shown as n(%) or mean±SD.

2. Age, BMI, Number of ribs fractured, Trauma to admission (days), Admission to intervention (days), Admission to discharge (days), and NRS of maximal pain after enrollment, were analyzed with One-way analysis of variance with Scheffé’s post hoc testing.

3. Gender, Current smoker, Mechanism of injury, Complications, and Chest tube/pig tail insertion, were analyzed with Chi-Squared test.
