# Supplementary material for: Acupuncture Analgesia in Patients With Traumatic Rib Fractures: A Randomized-Controlled Trial
Source: Front Med (Lausanne). 2022 May 27;9:896692. doi: 10.3389/fmed.2022.896692 (PMC9197317; doi:10.3389/fmed.2022.896692)
Supplement: Supplementary file 2 [file Table_2.DOCX]

**Supplementary Table 2. Pain intensity before and after intervention of each day, evaluated with NRS (using intention to treat analysis)**

| **NRS** | **Intervention day** | **Acupuncture** | **Laser acupuncture** | **Sham laser acupuncture** | ***p* value** |
| --- | --- | --- | --- | --- | --- |
| Average | pre D1 | 6.18±1.24 | 6.10±1.24 | 6.03±0.97 | 0.845 |
|  | post D1 | 5.20±1.27 | 5.05±1.18 | 5.65±1.12 | 0.067 |
|  | post D2 | 3.73±1.19**^*^** | 4.00±1.28**^*^** | 4.89±1.31 | P<0.001 |
|  | post D3 | 2.73±1.02**^*^** | 2.73±1.24**^*^** | 4.00±1.44 | P<0.001 |
| Deep breath | pre D1 | 5.25±2.23 | 4.72±2.57 | 5.15±1.76 | 0.532 |
|  | post D1 | 4.30±2.10 | 3.73±2.20**^*^** | 5.00±1.78 | 0.022 |
|  | post D2 | 3.00±1.78**^*^** | 2.95±1.82**^*^** | 4.16±1.83 | 0.006 |
|  | post D3 | 2.19±1.18**^*^** | 2.11±1.45**^*^** | 3.26±1.67 | 0.001 |
| Cough | pre D1 | 7.98±1.61 | 7.83±1.92 | 7.65±1.59 | 0.698 |
|  | post D1 | 7.55±1.57 | 7.13±2.23 | 7.53±1.63 | 0.511 |
|  | post D2 | 6.24±1.75 | 6.36±2.17 | 7.11±2.09 | 0.138 |
|  | post D3 | 5.46±2.09 | 5.27±2.47 | 6.43±2.32 | 0.078 |
| Turnover | pre D1 | 8.45±1.65 | 8.60±1.52 | 8.05±1.69 | 0.296 |
|  | post D1 | 7.90±1.78 | 7.93±1.75 | 7.80±1.88 | 0.948 |
|  | post D2 | 6.65±2.12 | 6.77±2.01 | 7.24±2.06 | 0.425 |
|  | post D3 | 5.62±2.13 | 5.35±2.65 | 6.54±2.33 | 0.090 |

*Significance when compared with sham laser acupuncture group, *p < 0.05.

Abbreviations: NRS, Numerical Rating Scale; pre D1, NRS score on day 1 before treatment; post D1, D2, D3, NRS score on day 1 to 3 after treatment
